# An Early Collaborative Intervention Focusing on Parent-Infant Interaction in the Neonatal Period. A Descriptive Study of the Developmental Framework

**DOI:** 10.3390/ijerph18126656

**Published:** 2021-06-21

**Authors:** Charlotte Sahlén Helmer, Ulrika Birberg Thornberg, Evalotte Mörelius

**Affiliations:** 1 Division of Nursing Sciences and Reproductive Health, Department of Health, Medicine and Caring Sciences, Linköping University, SE-581 83 Linköping, Sweden; e.morelius@ecu.edu.au; 2 Department of Behavioural Science and Learning, Linköping University, SE-581 83 Linköping, Sweden; ulrika.birberg.thornberg@liu.se; 3 School of Nursing and Midwifery, Edith Cowan University, Joondalup, WA 6027, Australia

**Keywords:** behavior, caregivers, descriptive design, early intervention, family health, infant health, infant premature, intensive care neonatal, neonatal nursing, parents

## Abstract

Moderate to late preterm infants are at risk of developing problems later in life. To support attachment and infants’ development, high quality parent-infant interaction is important. Parent-infant interaction is known to improve through intervention programs but since no such intervention program is addressed directly to moderate to late preterm infants, a tailor-made intervention was developed. The aim was to describe the rationale, development, framework and practical provision of a new early collaborative intervention program. This study has a descriptive design and the intervention is described using the Template for Intervention Description and Replication. During an intervention-session, the preterm infant’s cues are made visible to the parents as they perform an everyday care-taking procedure. Instant feedback is delivered to give the parents the opportunity to notice, interpret and respond to cues immediately. The infant’s response to the parent’s action is discussed in a dialogue to instantly guide parents´ awareness of the preterm infant’s subtle cues. This study describes a new early collaborative intervention, developed to support interaction between parents and their moderate to late preterm infants starting in the neonatal intensive care unit. Clinical studies evaluating parental experiences as well as the effects of the early intervention are ongoing, ClinicalTrials.gov NCT02034617.

## 1. Introduction

Parent-infant relations affect infant development [1]. Ainsworth described the need for the parent to be sensitive towards the infant’s cues and to read, interpret and respond to the cues prompt and accurately, in order to develop a well-functioning parent-infant interaction [2]. Parent-infant interaction is an act where the parent and the infant modify their behaviors depending on the feedback provided by one other [3]. A sensitive and responsive interaction between parents and their infants facilitate a secure attachment [4,5] and improve infant cognitive development [6].

The importance of early identification of risks in the parent-infant relationship, and a need to start supporting parents in the neonatal intensive care unit has recently been highlighted [7]. In neonatal care parent-infant relations are more vulnerable due to several factors which affect interaction patterns in families negatively. The cues of preterm infants are more subtle due to their immature neurological system [8], and thus more difficult to detect and respond to. Prolonged hospitalization, separation, parental distress, bonding difficulties and stress are other factors which hinder interaction [9,10,11].

Moderate to late preterm infants, are at a great risk of delays in physical and cognitive development [12,13,14,15]. The majority of the approximately 15 million infants born preterm [16] are born moderate or late preterm, between gestational week 32 and 37 [17,18], due to various reasons this group is constantly growing [19,20].

Intervention programs starting at the neonatal intensive care unit have previously shown a positive effect on infant development [21,22,23] and parent-infant relations [24,25]. Brazelton developed The Neonatal Behavioral Assessment Scale (NBAS), designed to explain the infant’s response to the environment and the infant’s communicative and interactive behavior [3]. This assessment scale has inspired several interventions aiming to improve parent-infant interaction and has been used as an intervention in itself. However, a recent review found low evidence in the effect of NBAS as an intervention to improve parent-infant interaction [26]. Nevertheless, the way Brazelton described infant behavior and social competence, is valued and of great importance for our understanding of parent-infant interaction.

To our knowledge, few early intervention programs have aimed to support parent-infant interaction for moderate to late preterm infants. One exception is a study by Ravn, who used the Mother Infant Transaction Program [27]. However, this program is extensive [28] which makes it difficult to incorporate with short hospital stays. Moreover, current research does not support improvements in cognition and behavior development among moderate to late preterm infants [29].

Most intervention programs focus on educating parents in their preterm infant’s behavior and needs whereas few programs aim to support the preterm infant simultaneously [24]. Intervention programs aiming to support relationships benefit by having a bidirectional focus, targeting both the preterm infant and the parents [30]. Few existing intervention programs for supporting parent-infant interaction are inclusive of both parents [24]. Therefore, psychosocial support along with engagement of both parents in the care has been suggested [31,32].

Our idea was to develop a feasible, tailored intervention program that could be incorporated with short hospital stays, a program for supporting parent-infant interactions starting at the neonatal intensive care unit for this large group of preterm infants and their parents. We developed the intervention program with the focus on sensitizing parents to the needs of the preterm infant and supporting parents’ interactions with their infants in a responsive way to enhance preterm infant wellbeing and development. At the same time the preterm infant was encouraged to express needs and thereby strengthen parental abilities. By introducing the intervention in the neonatal intensive care unit and continuing after discharge we bridge the gap between the hospital stay and the home. Our focus was on supporting the moderate to late preterm infant as well as the parents simultaneously, as they are all part of the interactional pattern in the family. The aim of this study was to describe the rationale, development, framework and practical provision of a new early collaborative intervention program.

## 2. Methods

The study project is registered in an international clinical trial register, ClinicalTrials.gov NCT02034617.

### 2.1. Design

This study has a descriptive design. The rationale, development, framework and practical provision of the EArly Collaborative Intervention (EACI) program are described using the Template for Intervention Description and Replication (TIDieR) [33] as a categorization matrix.

### 2.2. Setting

The neonatal intensive care unit where the early collaborative intervention has been developed is a 16-cot family-centered, level-three, single-room unit in Sweden.

The unit has an ongoing ambition to educate staff in evidence-based methods. One focus has been on certifying staff in the Newborn Individualized Developmental Care and Assessment Program (NIDCAP), based upon Heidelise Als´ synactive theory [34], an extension of the NBAS [3]. Assistant nurses, registered nurses and neonatologists have been trained and certified. As they have a certification in assessing newborn infant behavior and knowledge in how to support parents in caring for their preterm infant in a responsive way, seven of them also performed the early collaborative intervention program. The seven nurses and assistant nurses are called EACI-providers. Their competencies have also been used to develop the intervention program.

### 2.3. Standard Care at the Unit

Standard care for moderate to late preterm infants is provided by parents with support from registered nurses and assistant nurses. Parents are expected to stay at the unit day and night, together with their preterm infant and skin-to-skin care is routinely practiced. At the unit the parents have a kitchen for preparing meals with space to invite healthy relatives and friends, and siblings are welcome to stay with their family as well. As soon as the preterm infant is cardiorespiratory stable and is able to feed small amounts, the family is discharged from hospital to homecare, which includes checkups from a nurse twice a week. When the preterm infant can feed without the support of a feeding tube, the family is discharged from homecare.

### 2.4. Participants

Participants are families with moderate to late preterm infants at the neonatal intensive care unit. They usually have a rather short hospital stay and are in need of an early, flexible support incorporated during care. The parents are the primary caregivers during the hospital stay, and care is therefore dependent on the experiences and sensitivity of the parents.

### 2.5. Procedure

Families with moderate to late preterm infants are within the first three days after the preterm infant’s birth asked to be involved in the EACI program. Families are given information and those who consent are included. The coordinator nurse assigns an EACI-provider to provide the EACI according to the planned schedule.

### 2.6. Ethical Considerations

For the evaluation of the early collaborative intervention program, ethical approval was obtained from the Regional Research Ethics Committee, registration number 2013/367-31. Parents were asked for permission and provided written informed consent for publication of the written summary with photos, the Supplemental File S1.

## 3. Results

The theoretical framework of the early collaborative intervention program is based on the attachment theory and Mary Ainsworth’s early work on parent sensitivity [2,5]. A secure attachment is facilitated by high-quality parent-infant interaction [1,2]. To develop a well-functioning, parent-infant interaction the parent needs to be sensitive towards the infant’s cues and read, interpret and respond to the cues prompt and accurately. Thus, parental responsiveness is one of the core concepts of the early collaborative intervention. Additionally, the intervention is influenced by the NBAS, in explaining the infant’s response to the environment and the infant’s interactive behavior [3], making the infant cues and behavior visible for the parent and thereby possible to respond to. The start of the EACI program happens early after the preterm infant’s birth as an early start have shown to improve parent-infant relations [24].

The early collaborative intervention program is described according to the TIDieR checklist [33] below and in Table 1.

### 3.1. Item 1 the Name of the Intervention

The name is the Early Collaborative Intervention (EACI) program, early, as it starts within the first days of the moderate to late preterm infant’s life, collaborative, as both parents and the preterm infant are included in the guiding. The collaboration is defined as an integrated effort of the stakeholders to achieve a shared goal. Communication as well as interaction between the parents, the preterm infant and the providing staff is dependent on the feedback provided from one another. All participants during an EACI contribute to the interaction but focus is on the interaction between the parents and their preterm infant.

### 3.2. Item 2 the Rationale and Development

The rationale for developing the EACI program was experiences that individualized support provided after a NIDCAP observation were only provided for extremely preterm infants, omitting moderate and late preterm infants. There were also experiences of difficulties to communicate the infant’s cues and needs to the parents when not being able to talk to them during the observation, since this is not part of a NIDCAP observation. Furthermore, a hands-on guiding for parents of moderate to late preterm infants was thought to be a more appropriate approach as this group of preterm infants has reached a more robust development stage than the extremely and very preterm infants. Hence, the EACI was developed to support parent-infant interaction and establish equality among infants and parents using tailored support. Furthermore, to make parents and staff aware of moderate to late preterm infants´ variety of communication behavior, in order to improve a well-suited care thus achieving optimal infant development. Additionally, as the hospital stay becomes shorter, facilitating early discharge as preterm infants are cared for by their parents at home supported by neonatal homecare.

The EACI program was designed and developed in a Swedish neonatal intensive care unit, by a core-group consisting of staff certified in the Newborn Individualized Developmental and Assessment Program, a nurse co-director, a physician, a psychologist and researchers in developmental psychology and neonatal nursing. The core-group had earlier been involved in research on early interventions and had clinical and theoretical knowledge. To standardize performance of intervention regarding target group, starting point and intervals of the intervention, discussions were performed within the core-group until consensus was reached. The EACI-providers were assured working hours to deliver EACI-sessions in the hospital as well as in the families´ homes.

### 3.3. Item 3 Informational Materials

Focus during the provision of the EACI is on verbal communication. Still, some notes are taken by the EACI-provider, to later help the parents recall the session. The communication is based on the infants´ behavior and the parents´ interaction with their preterm infant and results in a short, written summary. The summary is usually one or two A 4 pages, including photos to further highlight different reactions or cues of the infant. Photos are especially important for parents with difficulties reading Swedish and parents who for any reasons have difficulties benefiting from a written document. The summary is included to the infants´ medical charts and a copy is given to the parents. In the summary the infant’s behavior and communicative activities is briefly described along with suggestions for the parents on how they can care for and interact with their preterm infant. An example of the short summary is provided in the Supplemental File S1. 

### 3.4. Item 4 the Procedure of the EA

One important goal is to support parents in their awareness of their preterm infant’s efforts to interact with the parents. By doing so the EACI-provider guides the parents to see, interpret and respond to the infant’s cues. Based on the infant’s alertness, the parents can for example be asked to come closer to the infant’s face facilitating opportunity for communication in a way suitable for both the infant and parents. The time of infant alertness may be short and the parents will have to adjust their communication not to overload the preterm infant immature neurological system.

Another goal during the EACI is to guide the parent-infant dyad by giving words to the infant’s behaviors and cues. The EACI-providers specifically guide the parents to acknowledge cues of distress, stability, breathing irregularities, grimaces, movements, tone, state-regulation, social and self-balancing capacity. Based on what the provider and the parents observe, the provider can give recommendations on how the environment around the premature infant and the care of the infant can be improved. For example, if the infant stretches and tries to brace his or her feet against something, the provider may ask the parents questions based on the behavior of the infant. Such as: What do you think your infant’s stretching behavior means? or How can you support your infant when she or he is stretching like this? The parents are therefore made aware of the infant’s efforts to self-regulate by attempting to support his or her feet. If not noticed by the parents, the EACI-provider shows and describes how the parents can assist the behavior by supporting the infant’s feet with, for example their hands. Questions are not only to help the parents focus on the needs of the preterm infant but also to help the parents see the cues by themselves, and thereby feel more confident and secure in taking care of their preterm infant. The infant’s response to the care is immediately communicated by words and gestures by the provider to the parents, and together the provider and parents give words to the behavior of the infant. The focus is also on encouraging parents to acknowledge the infant’s current developmental stage and to recognize and be prepared for the next developmental steps.

### 3.5. Item 5 the Providers

To increase reliability, the EACI was delivered only by staff certified in the Newborn Individualized Developmental and Assessment Program. To gain a certification, education together with systematic behavioral observations supervised by a trainer is mandatory. Focus is on learning the preterm infant’s behavioral language and the establishment of a relationship-based care, instead of a task-oriented care. In line with this, the training raises the importance of emotional availability towards the infant as well as the parents [35]. The emotional availability construct is intended to capture the degree to which both the parent and the infant are accessible and able to respond appropriately to the other’s emotional signals [36]. To ensure competence, the EACI providers had been NIDCAP-certified for 3 to 12 years, (mean 6.8 years) and had between 15 to 40 years of experience working in the neonatal intensive care unit.

### 3.6. Item 6 the Delivery

The EACI is delivered with both parents present at a time chosen by them. If for any reason only one parent is present, the EACI-provider describes the outcome of the EACI to the other parent as soon as convenient for the family. The parents´ perceptions, descriptions and emotions regarding their infant are valuable contributions to the understanding of the family situation, and parents are encouraged to share their views of their preterm infant. It is the needs of the infant and the parents as well as paying respect to their situation, knowledge and previous experiences that are the guiding principles. The intervention is delivered for one family at a time, by one provider at a time. A coordinator nurse at the unit organizes for a provider to be able to provide the family with an EACI within the timeframe. The seven EACI-providers were providing the intervention at the hospital as well as in the families´ homes. Different EACI-providers may provide the same family with the interventions at different occasions. The choice of EACI-provider is dependent on who is scheduled for work. The EACI-sessions can take place during breastfeeding, a diaper change, a bath or other routine care-taking procedures.

### 3.7. Item 7 Where the EACI Was Delivered

The first and second EACI-sessions are delivered at the hospital and the third in the family’s home. Each EACI-session is delivered in a natural setting in the room where the family stays, whether it is an intensive care room or a family-room at the unit or in the family’s home.

### 3.8. Item 8 When and How Much

**Figure 1:**
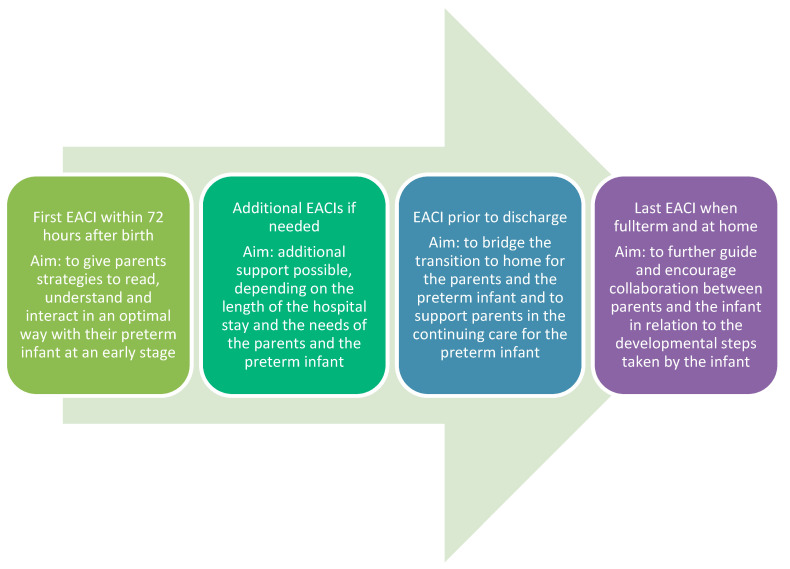
Flowchart of the EACIs and a description of the content during each session.

At least three EACI-sessions are delivered, at a time appropriate to both the parents and the preterm infant. However, the first EACI should be delivered within 72 h after birth and the second within 48 h before the family leaves the hospital. The EACI-sessions in the hospital requires a minimum of preparation once the EACI-provider is familiar with the intervention. As there is a need for the parents to be able to recognize their preterm infant’s development and customize their care both in hospital and after discharge, the third EACI-session is delivered after discharge, when the premature infant is full-term, Figure 1.

### 3.9. Item 9 Tailoring of the EACI

Depending on the feedback from the preterm infant and the parents, the provider may need to adapt the intervention. This adaption may consist of additional EACI-sessions, a necessity due to the family’s situation and length of hospital stay. The length of the EACI may differ. Usually the delivery of one EACI-session takes about one to two hours, since it is the family’s needs that determine how much time the providers spend with each family. The time spent delivering each EACI-session also depends on the alertness of the preterm infant, the parents´ skills and whether the EACI-provider and the parents acquire the opportunity to capture the preterm infant’s needs and competences.

### 3.10. Item 10 Modifications of the EACI

There is a flexibility incorporated in the EACI, since the interaction between the EACI-provider and the parents and the feedback from the parents are taken into consideration when delivering the EACI. Depending on the feedback from parents and the infant, the provider may need to modify the support to suit their current individual needs.

### 3.11. Item 11 Intervention Adherences of the EACIs

At the start of delivering the intervention program recurrent discussions within the core group were performed to bring consensus on the provision as well as the written summary. When the EACI-providers became used to working with the EACI, discussions and support were ongoing within the provider group.

### 3.12. Item 12 to the Extent EACI Was Delivered as Planned

The aim was to deliver every family with at least three EACI-sessions. For the time being, 196 EACI-sessions have been delivered for 70 families. Out of these families 45 have received three sessions and seven families have received four or five sessions due to an extensive hospital stay or a need for the extra guidance (as described in item 9). Fourteen families have had two sessions. Remaining four families have received less sessions. Reasons for not having all the three EACI-sessions could be a very short hospital-stay for the family, lack of qualified staff or an unexpected transfer to another hospital due to lack of intensive care beds.

## 4. Discussion

This study describes a new early collaborative intervention program. It is developed to be used in the neonatal intensive care unit to support parents and their moderate to late preterm infants, as there is a lack of an intervention program designed for this group. Fundamental to this program is that a sensitive and responsive parental behavior is a prerequisite for a well-functioning parent-infant interaction which in turn, facilitates a secure attachment and optimized cognitive development for the infant.

The EACI program strives to empower and support parents to interact with their infant and to strengthen emotional mutuality. The guiding is based on the knowledge of Ainsworths´ work on how parents need to act sensitive towards infant’s cues in order to establish a well-functioning interaction [2]. Early interventions starting in the neonatal intensive care unit, have previously shown to improve parent-infant relations [24]. However, several interventions are more extensive than the EACI program [24]. For example, the Mother Infant Transaction Program improves preterm parent-infant interaction [27] but the extensiveness of the program makes it difficult to incorporate with short hospital stays, as in our setting. Since there are time and economic constraint in public healthcare, it is important to find a feasible but still effective intervention program. The EACI program is delivered without preparation and the written summary after the session is short but still, an important addition to the dialogue. The written summary serves as an objective report that the parents can bring home as a complementary support after a short hospital stay. The objective was to provide every family with three EACI-sessions. One reason for delivering a reduced number of sessions is a lack of intensive care beds on the unit which has been described as a limiting factor in the neonatal context [37].

The EACI is provided through an individual, mutual discussion in a dialogue manner, focusing on both parents and infant simultaneously. This builds upon previous research showing that intervention programs targeting the relationship between preterm infants and their parents should be individualized [31,38] and target both the preterm infant and the parents [30]. Preterm birth impacts the expected new role as a parent [39]. This is not just for the mothers; the interactive paternal-preterm infant pattern has also shown to affect preterm infant development [40]. A father active in caretaking has a positive effect on maternal-preterm infant interactive behavior [41]. It has been suggested that paternal negative feelings of the mother-preterm infant interactive behavior in turn have a negative effect on the mother’s interactive behavior [42], while including fathers in interventions in the neonatal intensive care unit can facilitate the spouse relationship [43], pointing at the importance of supporting both parents as they are part of the interactive pattern in the family.

Support has also been suggested to be effective when made into a collaboration between parents and staff [44], based on the preterm infant’s cues [25,45]. EACI offers a way to support parent-infant relations as the provider explains the preterm infant’s subtle cues in order to help parents feel secure in caring for their infant. Preterm infant subtle cues are often difficult to notice and respond to and may be misunderstood as an infant averting behavior. Understanding the cues will also help parents enjoy their preterm infant and thereby strengthening the relationship further. Mothers have previously described how a well-functioning interaction with their late preterm infants improved their feelings of motherhood [46].

Parents in the neonatal context have previously highlighted how well-functioning communication with staff is of great importance in managing the stressful situation of being a parent to a preterm infant [47], and how the communication can guide them in understanding their preterm infant’s relational needs [48]. During the EACI, the parents´ perceptions, descriptions and emotions regarding their infant are valuable contributions to the understanding of the family situation. Parents are encouraged to share their view about their preterm infant and the communication between the parents and the EACI-provider is an important part of the collaboration. This is in accordance with the Mother Infant Transaction Program [28]. However, where the Mother Infant Transaction Program uses the reflective dialogue to focus solely on the parent in caring for the infant, the EACI have a bidirectional aim, focusing on both the parent and the preterm infant. 

It is important to pay attention to all preterm infants in the neonatal intensive care unit, regardless of gestational age, as delays in physical and cognitive developmental outcomes are evident even for moderate and late preterm infants [12,49]. It is vitally important to involve parents in the care, as this is recommended for optimal development [35,50]. Moderate and late preterm infants often have a shorter hospital stay, compared to very and extremely preterm infants. This puts a large responsibility on the parents in being able to recognize and respond to their preterm infant in a suitable way after discharge. The EACI program may offer a way of supporting parents and preterm infants. Moreover, since moderate to late preterm infants constitute a large group in the neonatal care, the EACI program offers great opportunities to improve development and parental confidence in many families otherwise left without specific support. The EACI program brings equality among preterm infants and their parents in an intensive care context where otherwise much focus is on extremely preterm infants [51].

This study gives no evidence of if the EACI program can increase parent-infant interaction. Further studies are needed to objectively evaluate the program from a staff and parent perspective as well as to report outcomes from an ongoing randomized controlled trial. In the study project, follow-ups are performed at one, four and twelve months and at four years since more longitudinal follow-ups are required to study long-term effects [52]. At one and four months parent-infant interaction is videotaped and will later be analyzed using two different instruments, the Ainsworth’s Maternal Sensitivity Scales including four subscales [53] and the Emotional Availability Scales, including six subscales [54]. Outcomes regarding, breastfeeding, parental depressive and anxiety symptoms, parental experiences of infant socio-emotional behavior and salivary cortisol will also be evaluated. Furthermore evaluation of factors known to be affected from parent-infant interaction and intervention programs [44] will be made. Infant cognition, language, motor skills and behavior will be assessed at twelve months with The Bayley Scales of Infant and Toddler Development-III [55]. At four years of age the child will be assessed with Wechsler Preschool and Primary Scale of Intelligence-IV [56] to tap cognition, language, visual-spatial skills and memory. Parents perceived stress, perceived parental functions will be evaluated by questionnaires.

### Strengths and Limitations

This new early intervention program fills a gap, as it focuses on moderate to late preterm infants that usually have a rather short hospital stay. It is developed in collaboration with staff in the neonatal intensive care unit, and provides immediate, individualized, reflexive support to both parents and preterm infants simultaneously. The intervention is grounded by an interdisciplinary group of clinicians and researchers in collaboration with staff from the neonatal intensive care unit.

Another strength of this paper is the thorough description of the EACI program according to the TIDieR [33].

However, one limitation is that at this stage only a small group of seven nurses with a certification in the NIDCAP are performing the EACI-sessions. The purpose for this was to ensure quality of the delivered program during the randomized controlled trial. Further studies will evaluate if the EACI program can be provided by staff without the certification, with maintained quality.

## 5. Conclusions

This study describes a new early collaborative intervention program for moderate to late preterm infants and their parents, founded in the attachment theory. The objective is to improve parent-infant interaction which in turn will facilitate attachment and cognitive development of the infant.

## Figures and Tables

**Table 1:** TIDieR items and description of the EACI.

TIDieR Item	Content	Key Components of the EACI
1	The name	The EArly Collaborative Intervention, the EACI program
2	(a) The rationale	To support parents and preterm infants in a tailored individualized way To support parent-infant interaction in the NICU To establish equality among every parent and preterm infant To improve a well-suited care and thus achieve the best possible infant development To facilitate early discharge for preterm infants and parents
	(b) The development	In a Swedish NICUD eveloped and standardized by a core-group
3	Informational materials	Primarily hands-on guidance with a short summary report
4	The procedure	Parent support in awareness of the infant’s attempt to interact Giving words to the cues of the preterm infant Instant feedback and guidance during active parental involvement
5	The providers	NIDCAP-educated and certified
6	The delivery	Both parents included Collaboration between infant, parents and staff One family at a time One provider at a time A guided practice during natural care-taking procedures
7	Where the intervention occurred	In a natural setting In the hospital and in the family’s home when discharged
8	When and how much	Three times: within 72 h after birth, within 48 h before discharge and when full-term When the family decides it is feasible
9	Tailoring of the intervention program	Additional EACI-sessions if needed The family’s needs are the guiding principle Adaptions in terms of length for each EACI-session
10	Modifications	Flexibility is incorporated due to the families’ individual needs
11	Intervention program adherence or fidelity assessment	Considerations on adherence of the provision of the EACI as well as the written summary
12	To the extent the intervention program is delivered	Deviations may occur if the family is discharged or transferred to another hospital

EACI = EArly Collaborative Intervention, NICU = Neonatal Intensive Care Unit, NIDCAP = Neonatal Individualized Developmental Care and Assessment Program, TIDieR = Template for Intervention Description and Replication.

## Data Availability

Not applicable.

## Supplementary Materials

The following are available online at https://www.mdpi.com/article/10.3390/ijerph18126656/s1, Supplemental File S1, EACI of a preterm infant.
