# Supplementary material for: An Early Collaborative Intervention Focusing on Parent-Infant Interaction in the Neonatal Period. A Descriptive Study of the Developmental Framework
Source: Int J Environ Res Public Health. 2021 Jun 21;18(12):6656. doi: 10.3390/ijerph18126656 (PMC8296427; doi:10.3390/ijerph18126656)
Supplement: Supplementary file 1 [file ijerph-18-06656-s001.zip › ijerph-1248933-supplementary.pdf]

2019-01-25

EACI no. 2

The baby boy was observed for just over an hour around lunchtime, in interaction with his mother and father. One of his nurses was on hand for the family in the room. The baby boy had his diaper changed, was bathed for the first time and after that, had his meal.

Today the boy demonstrated how he could support himself, when he had support from his parent´s hands, the bed and the blanket around him. Then he managed to lie on his back. He held onto a finger or his own cheek and supported his feet against whatever was available in his attempts to keep his body stable during the diaper change and the bath. During the bath he looked around attentively, following his parent´s voices. He showed his hunger and that he wanted to breastfeed by sucking intensely on the pacifier and moving his hand to his mouth. He nursed for a while at the beginning of the feeding tube meal repeatedly sucking strongly.

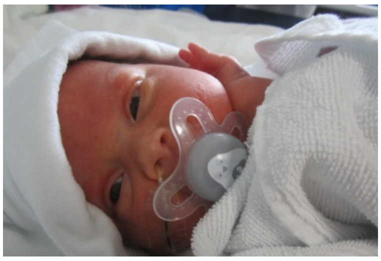

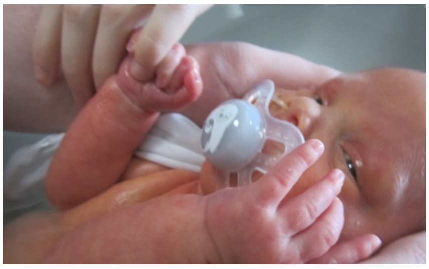

He is calm when he gets to hold on to something.

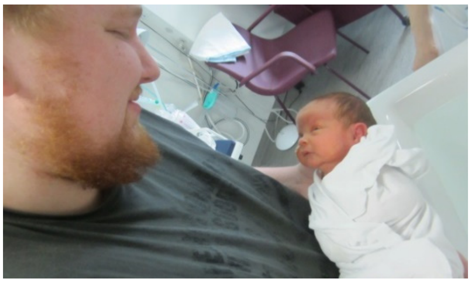

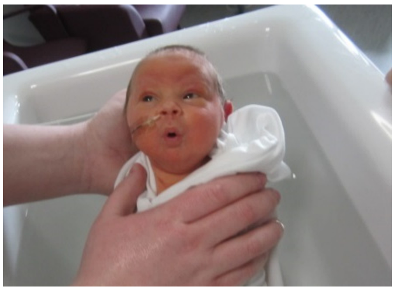

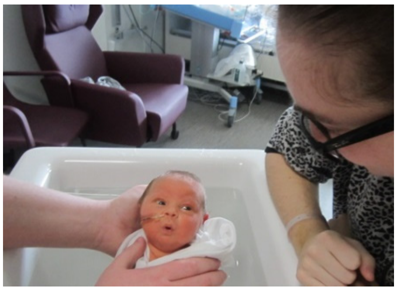

He listens and looks attentively at his mother and father.

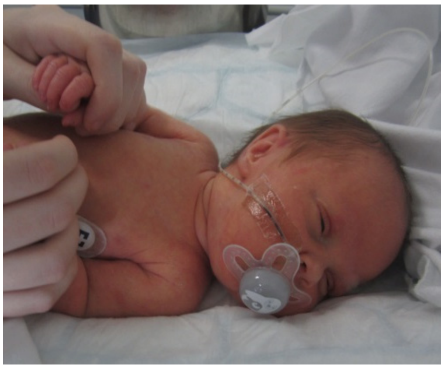

He squints to keep the light out.

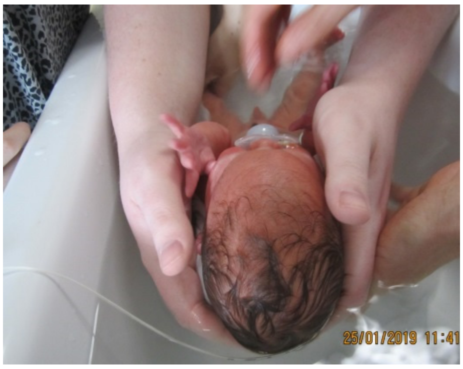

He flails his hands and legs when the support from the blanket disappears, despite his father´s secure hands.

2019-01-25

Suggestions and ideas regarding how you can help your baby boy in his attempts to find balance and reach a calm state.

Continue to let him be close to you, because then he becomes calm and relaxed. Consider if it would be convenient to have a kangaroo top – they can offer more freedom to move around the room while keeping him close.

Continue to support him with your hands and a blanket, because he has so clearly shown that this enables him to be balanced and calm. Both in the bath, and when moving him around.

Your baby boy showed clearly, by getting sad and stretching his arms and legs out, when he needed a little break to get stable again. Continue to let him do this by being as calm and gentle as you were when you cared for him. This helps him to use his energy for staying awake, interacting with you and being able to breastfeed when he is hungry.

Consider if it is easier for him to be slightly on his side when his diaper is being changed and when drying his bottom. This way his legs are not pressed onto his stomach in the same way, and he can breathe more efficiently.

Continue to watch for his signals of hunger. Let him breastfeed at those times. Trust that he knows when he is hungry, even if it isn't feeding time according to the clock.

Continue to talk and listen to your little boy, and you will get to know each other more and more every day.

Thank you for allowing me to spend some time with you today.

Name

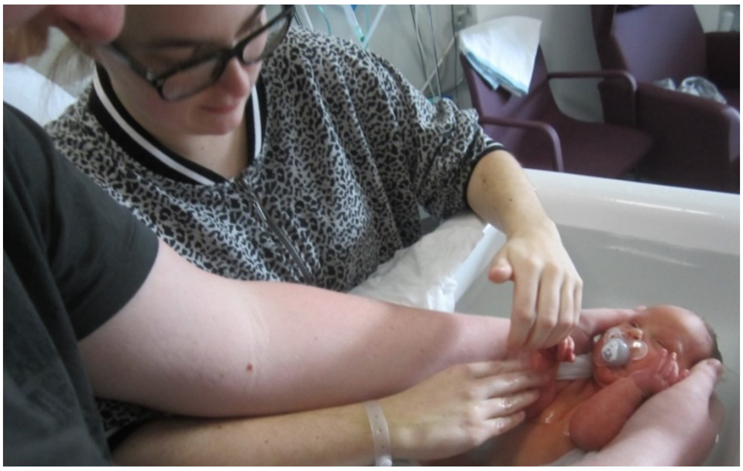

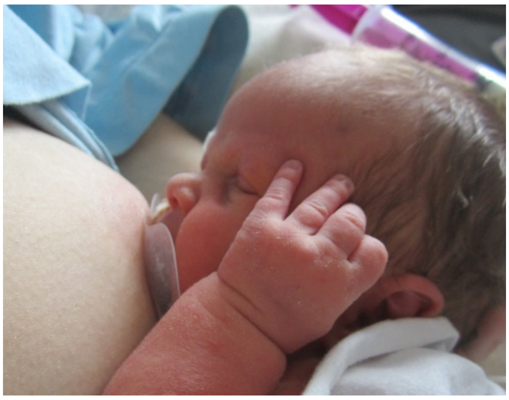

He falls asleep well after nursing
